# The RACE-3 is on: double-locking sinus rhythm by upstream and downstream therapy

**DOI:** 10.1093/eurheartj/ehy018

**Published:** 2018-02-07

**Authors:** Davor Pavlovic, Paulus Kirchhof, Larissa Fabritz

**Affiliations:** 1 Institute of Cardiovascular Sciences, University of Birmingham, Birmingham, UK; 2 Sandwell and West Birmingham NHS Trust, Birmingham, UK; 3 University Hospital Birmingham NHS Foundation Trust, Birmingham, UK

## Abstract

**Figure:**
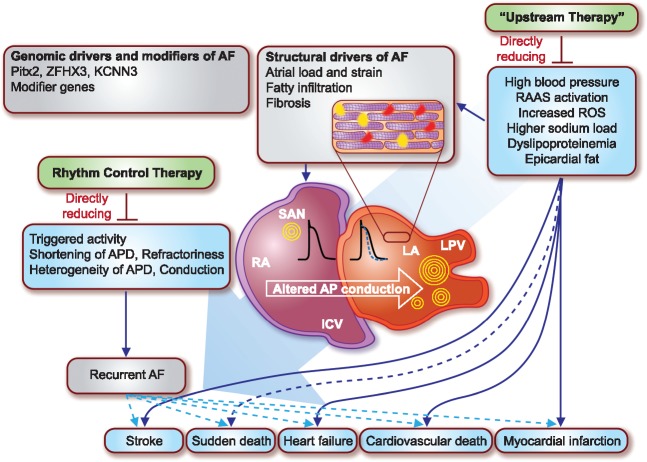

**This editorial refers to ‘Targeted therapy of underlying conditions improves sinus rhythm maintenance in patients with persistent atrial fibrillation: results of the RACE 3 trial’^†^, by M. Rienstra *et al.*, on page 2987.**

Recent advances in the field of anticoagulation have given us powerful tools to reduce stroke and its associated disease burden in patients with atrial fibrillation.^1–4^ More work needs to be done to offer adequate anticoagulation to all patients with atrial fibrillation at risk for stroke,^5^^,^^6^ and ongoing trials explore the limits of anticoagulation in patients with very low levels of atrial arrhythmias.^7^^,^^8^ However, even in adequately anticoagulated patients with atrial fibrillation, important unmet therapeutic needs remain, particularly around prevention of sudden death, heart failure, and unplanned cardiovascular hospitalizations.^5^

Many groups have speculated that ‘upstream therapy’ or ‘prevention of atrial remodelling’ can improve rhythm control therapy in patients with atrial fibrillation.^9^^,^^10^ Clinical trials conducted so far have not conclusively demonstrated effectiveness of either angiotensin-converting enzyme inhibitors (ACE-Is), angiotensin receptor blockers (ARBs), or statins in reducing recurrent AF.^11–13^ In this issue of the journal, van Gelder and colleagues report the outcome of the RACE-3 study.^14^ RACE-3 tested whether the addition of a comprehensive ‘upstream therapy’ package, consisting of mineralocorticoid receptor antagonists (MRAs), statins, ACE-Is and/or ARBs, and cardiac rehabilitation including physical activity, dietary restrictions, and counselling, improves sinus rhythm maintenance in anticoagulated patients with persistent atrial fibrillation undergoing rhythm control therapy.^9^ This elegant design combines several important components of ‘upstream therapy’ into a single intervention, thus quantifying the positive effect of ‘upstream therapy’ in its totality for recurrent atrial fibrillation in 1 year.^15^

In RACE-3, patients randomized to the ‘upstream therapy’ intervention had lower blood lipid levels, lower levels of brain natriuretic peptide (BNP), and lower blood pressure than the control group at follow up, demonstrating that the intervention had the desired biological effects. The feasibility of an intervention to reduce the cardiovascular risk profile in patients with atrial fibrillation is an important finding in itself and should empower primary and secondary prevention initiatives. The authors found a slightly higher number of patients in sinus rhythm after 1 year, with a nominally significant *P*-value (*P* = 0.04) in the primary outcome of the study, defined as sinus rhythm on at least 6 out of 7 days of a 7 day Holter ECG at 1 year follow-up. Other rhythm outcomes were not different between groups, e.g. the number of repeat cardioversions, the time to recurrent atrial fibrillation, or cardiovascular hospitalizations. This may be due to the weaker long-term and indirect effects of the intervention on atrial electrical function (*Take home figure*) which contrasts with the immediate direct effects of antiarrhythmic drugs and ablation procedures. Of note, the use of ACE-Is/ARBs was high in both study groups, and catheter ablation was rarely used in the study population, with only seven ablations performed.

**Take home figure:**
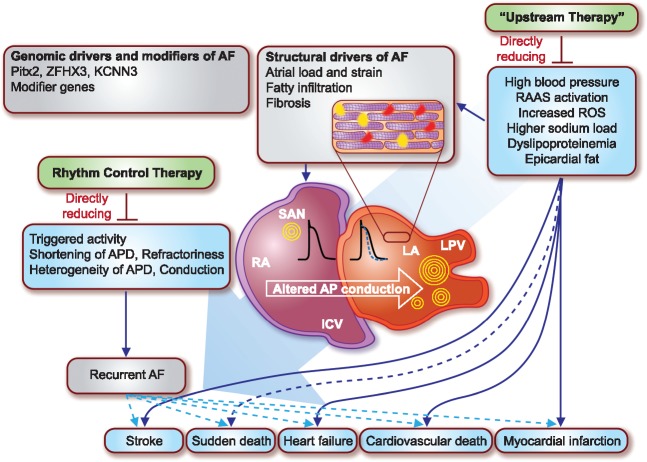
Illustration of major upstream and downstream drivers of atrial fibrillation (AF) and direct and indirect links to cardiovascular outcomes. Upstream therapy targets major indirect drivers of AF, including high blood pressure, renin-angiotensin-aldosterone-system (RAAS) activation, reactive oxygen species (ROS), increased sodium load, dyslipoproteinaemia and epicardial fat. Upstream therapy thus also reduces atrial load and strain, fibrosis and fat infiltration in the atria. Rhythm control therapy directly targets triggered activity, action potential duration (APD) shortening and slowed conduction across the atria. Inset; atrial electrical function can be altered by fatty deposition (shown in yellow) and interstitial fibrosis (shown in red). Dashed lines indicate less established links, solid lines established links to a delta of cardiovascular complications. ICV, inferior caval vein; LA, left atrium; LPV, left pulmonary vein; RA, right atrium; SAN, sinoatrial node.

The results illustrate two main points: (i) a comprehensive ‘upstream therapy’ treatment package in patients with persistent atrial fibrillation and some degree of heart failure only slightly improves prevention of recurrent AF in the short term; and (ii) such treatment seems safe and leads to desirable reductions in lipid profiles, BNP, and blood pressure.

## What does the study add?

Like every well-designed study, RACE-3 provides important answers and raises new questions. MRAs, statins, ACE-Is/ARBs, and cardiac rehabilitation improved important surrogates for cardiovascular outcomes without major safety concerns. As such, the study results demonstrate the feasibility of comprehensive cardiovascular risk reduction in patients with atrial fibrillation, supporting the concept of integrated care for these patients,^1^^,^^5^^,^^6^ as the authors discuss elegantly.^14^

RACE-3 also illustrates the limited short-term effectiveness of ‘upstream therapy’ for preventing recurrent atrial fibrillation after cardioversion: even a comprehensive package tackling underlying cardiovascular conditions by rehabilitation, statins, MRAs, and renin–angiotensin–aldosterone system (RAAS) inhibition did not affect the number of repeat cardioversions, time to recurrent atrial fibrillation, or cardiovascular hospitalizations. More efficient weight loss strategies could possibly also lead to better outcomes in the future, as there was only a slight decrease in body mass index in the intervention group in RACE-3. Longer term assessment of the intervention tested in RACE-3 may provide further benefits to the patients as the ‘upstream therapy’ package may have more pronounced effects on atrial fibrillation after several years of treatment. We look ahead for the long-term follow-up of this patient cohort for answers to these questions.

## What does that mean for clinical practice?

MRA inhibition, RAAS inhibition, and statins should be considered in patients with persistent atrial fibrillation as part of an integrated approach to the care of patients with atrial fibrillation.^5^^,^^6^ The results also illustrate that the effect of ‘upstream therapy’ on recurrent atrial fibrillation in patients with persistent atrial fibrillation is modest at best, and clearly weaker than the short-term effect of antiarrhythmic drug therapy or catheter ablation.^21^^,^^22^ Testing the effectiveness of ‘upstream therapy’ over a longer time frame may still demonstrate that such treatments lead to better outcomes. Nevertheless, targeted and direct treatment of electrical drivers of AF is needed to improve rhythm control therapy, e.g. early rhythm control interventions,^18^^,^^23^ hybrid therapy incorporating catheter ablation and antiarrhythmic drugs,^1^^,^^16^^,^^17^ and treatment approaches based on the major drivers of atrial fibrillation (*Take home figure*).^15^

## More upstream and downstream work is needed

The results of RACE-3 illustrate that risk factor management cannot replace direct treatment of the electrical drivers of atrial fibrillation by antiarrhythmic drugs and catheter ablation (*Take home figure*). While we await the full publication of the CASTLE-AF trial outcome, the next few years should provide new information on the role of modern and comprehensive rhythm control therapy for cardiovascular outcomes in patients with atrial fibrillation.^16–18^ In addition, there is a clear need to improve rate control therapy to avoid worsening of heart failure in patients with atrial fibrillation, including mechanistic work to identify patients who benefit from specific treatments.^19^^,^^20^

Clearly, the road to successful maintenance of sinus rhythm requires careful consideration of the major health modifiers causing atrial fibrillation. A substantial body of evidence demonstrates that atrial fibrillation and other underlying cardiovascular conditions alter structural and electrical properties of the atria,^10^^,^^15^ including interstitial fibrosis, increased formation of extracellular matrix, alterations in cell–cell contact proteins, adipose tissue activation and infiltration, changes in gene expression pattern, oxidative stress, calcium abnormalities, and others. Dysregulation of the RAAS and autonomic dysfunction are found in atrial fibrillation, hypertension, heart failure, kidney dysfunction, or obesity, and further promote atrial remodelling. Early-onset atrial fibrillation in particular can be driven by a genetic or genomic component that must also be taken into consideration during treatment. Attenuation of such complex pathophysiological stimuli requires a collaborative effort of basic and clinical arms of our research, if we are to tackle the ever-increasing incidence and prevalence of atrial fibrillation.

## The double lock

Joint upstream and downstream therapy can provide a double lock to slow progression of the atrial fibrillation, but more work needs to be done. The results of RACE-3 thus call for a full-scale effort to tighten rhythm control therapy upstream and downstream, considering the major drivers of recurrent atrial fibrillation in patients by stratified therapy.

## Funding

This work was partially supported by the European Union [grant agreement no. 633196 (CATCH ME) to P.K. and L.F.], the British Heart Foundation (FS/13/43/30324 to P.K. and L.F., PG/17/55/33087 to D.P.), and the Leducq Foundation to P.K.

**Conflict of interest:** P.K. receives additional research support from the Medical Research Council (UK), the German Centre for Cardiovascular Research, and from several drug and device companies active in atrial fibrillation, and has received honoraria from several such companies. P.K. and L.F. are listed as inventor on two patents held by the University of Birmingham (Atrial Fibrillation Therapy WO 2015140571, Markers for Atrial Fibrillation WO 2016012783).

## Footnotes

^†^ doi:10.1093/eurheartj/ehx739.
